# Detecting large deletions at base pair level by combining split read and paired read data

**DOI:** 10.1186/s12859-017-1829-z

**Published:** 2017-10-16

**Authors:** Matthew Hayes, Jeremy S. Pearson

**Affiliations:** 1 Xavier University of Louisiana, 1 Drexel Dr, New Orleans, 70125 LA USA; 2 Department of Computer Science, Tennessee State University, 3500 John A. Merritt Blvd., Nashville, 37221 Tennessee USA

**Keywords:** Deletions, Structural variant, Sequencing

## Abstract

**Background:**

Genomic structural variants (SV) play a significant role in the onset and progression of cancer. Genomic deletions can create oncogenic fusion genes or cause the loss of tumor suppressing gene function which can lead to tumorigenesis by downregulating these genes. Detecting these variants has clinical importance in the treatment of diseases. Furthermore, it is also clinically important to detect their breakpoint boundaries at high resolution. We have generalized the framework of a previously-published algorithm that located translocations, and we have applied that framework to develop a method to locate deletions at base pair level using next-generation sequencing data. Our method uses abnormally mapped read pairs, and then subsequently maps split reads to identify precise breakpoints.

**Results:**

On a primary prostate cancer dataset and a simulated dataset, our method predicted the number, type, and breakpoints of biologically validated SVs at high accuracy. It also outperformed two existing algorithms on precise breakpoint prediction, which is clinically important.

**Conclusion:**

Our algorithm, called Pegasus, accurately calls deletion breakpoints. However, the method must be extended to allow for germline variant filtering and heterozygous deletion detection.

The source code that implements Pegasus can be downloaded from the following URL: http://github.com/mhayes20/Pegasus.

## Background

There are several underlying causes for the onset of cancer, but genomic abnormalities play a significant role in susceptibility to the disease. These abnormalities may include single nucleotide polymorphisms (SNPs) or small indels of a few base pairs (bp) in length. However, large scale genomic structural variants also play a role [1, 2]. These variants, which are typically larger than 1000 bp, include insertions, deletions, translocations, inversions, and tandem repeats [3, 4]. Structural variants (SV) can have deleterious effects on the health of an individual. If a SV occurs in or near a gene, it could adversely affect the intended function of that gene. An example of this phenomenon is the deletion of a tumor suppressing gene, or the amplification of an oncogene. Structural variants can also create fusion genes, which may code for proteins with cancer-causing effects. These fusions can be caused by translocations, inversions, or deletions [5, 6].

The impact of SVs necessitates the development of efficient methods to locate and characterize them. Many computational methods for this problem use data generated from next-generation sequencing (NGS) platforms. Methods for SV detection typically use either 1) abnormally mapped read pairs (or discordant pairs), or 2) single anchoring reads (or split reads). BreakDancerMax (BreakDancer) finds clusters of abnormally aligned read pairs (or discordant pairs), and it calculates the probability of each cluster based on a Poisson model [7]. Another program, called Delly [8] identifies structural variants by identifying abnormally mapped read pairs that are proximal to single-anchoring reads. The program then realigns these split-reads to identify precise boundaries. Several other algorithms exist to address the problem of finding structural variants in the genome [9–13].

We present a method, Pegasus, that finds groups of anomalously-mapped read pairs, and then subsequently aligns the *soft-clipped* portion of local reads to the reference, which could indicate a SV boundary. Pegasus had high sensitivity on a primary prostate cancer dataset and a simulated dataset. It also outperformed two other methods, Delly and BreakDancer, in breakpoint accuracy prediction. We previously presented an algorithm called Bellerophon that applied a similar approach to identify translocations [14].

## Methods

**Fig. 1:**
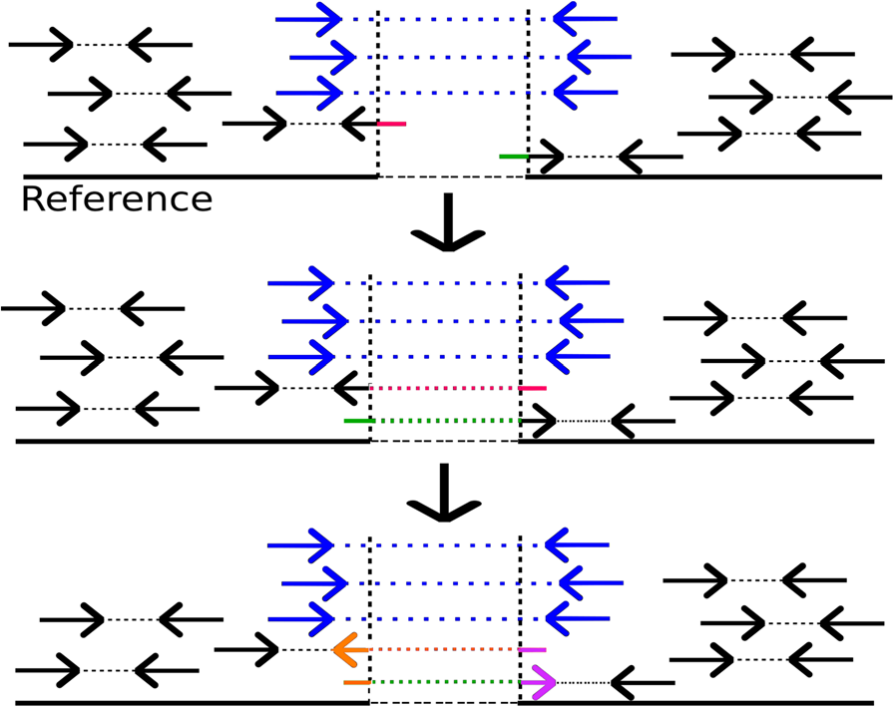
Visual depiction of Pegasus algorithm. The images show read pairs that are mapped to a reference genome, where the blue read pairs span a deletion event. (Top panel) Soft-clipped reads (pink and green subreads) are initially unaligned since they fall within the deleted region. Pegasus first clusters discordant read pairs that may indicate a deletion. It then maps the clipped subreads back to the reference using BLAT (middle panel). The subreads align to each side of the deletion event. The coordinate locations with the most aligned subreads are the predicted deletion breakpoints (bottom panel)

At the beginning of our pipeline, sequence reads representing a test (or donor) genome must first be aligned to the human reference genome. Our method takes as input a set of sorted alignment results in the SAM format [15]. In our experiments, we used BWA [16] to perform the alignment, though any alignment program can be used as long as it produces soft-clipped reads. Soft-clipped reads are reads that partially align to the reference; the unaligned portion of the read is still kept in the SAM record. Single reads that span a variant boundary are likely to be portrayed as soft-clipped in the alignment record, since the subread that lies within the variant will likely not align to the reference. As shown in Fig. 1, when a single read spans a variant boundary, the read only partially aligns to the reference, while the unaligned portion ostensibly belongs to the variant. After performing sequence alignment, Pegasus extracts all long *discordant* read pairs and soft clipped reads. The discordant read pairs are those that are abnormally aligned to the reference; their mapping is somehow different than what is expected. They indicate likely structural variants, which for our program are deletions. The soft-clipped reads are then used by our program to perform precise breakpoint refinement prediction. Specifically, for soft-clipped reads that are nearby to discordant read pairs, the clipped sub-read is realigned separately to find precise variant coordinates.

### Discordant read pair clustering and SV prediction Algorithm

To find likely variants, Pegasus must first find groups of discordant read pairs that could indicate a deletion, as shown in Fig. 1. The method defines a discordant pair as having a mapped distance between read pairs that is greater than *L*=*mean*+*k*∗*stdev*, where *mean* is the mean mapped distance between mates, *stdev* is the standard deviation of mapped distance lengths, and *k* is a user-defined parameter, which for Pegasus is 4 by default. We also require that when aligned to a reference genome, the relative orientation of the read pairs must be the same as it was during sequencing. For Illumina paired-end sequencing technologies, one read is sequenced from the forward (+) strand (relative to the p-arm telomere), while the other read is sequenced from the reverse (-) strand (relative to the q-arm telomere). Thus, most normally-mapped read pairs have forward-reverse orientation, where the read closest to the p-arm telomere has forward orientation. For deletion events, the relative orientation of the mapped reads will likely remain unchanged for non-complex deletion variants. Else, the variant could indicate a possible inversion or tandem repeat, which is not considered here.

The program takes read alignment results in SAM format and looks for clusters of overlapping discordant pairs such that the left reads in each cluster are within *L* base pairs of each other, and the right reads are also within *L* base pairs of each other. This is a necessary requirement because if a group of discordant pairs imply a true deletion event, then their mapped distances should be similar. When a group of overlapping discordant pairs is found, the program then searches for soft-clipped reads that are presumably near the SV breakpoint of the reads on either side of the potential deletion. It then extracts the soft-clipped portion of at least one read and realigns it to the reference genome using BLAT [17]. Compared to the size of the reference genome, the size of the cluster region (a few hundred bases) is smaller by several orders of magnitude. Because of this, it is unlikely that even a single clipped subread will realign to the region by chance.

To be predicted as a structural variant, a cluster of overlapping discordant pairs must satisfy two criteria: 1) there must be at least *minD* discordant read pairs in the cluster, which for Pegasus is 3 (by default), and 2) there must be at least *minS* soft-clipped reads from either side of the event that remap within the cluster region, which is the region from the outermost read in the cluster towards the variant breakpoint. For Pegasus, this value is also 3 by default.

#### Preliminaries

Let *R*(*p*) denote the set of reads in a discordant read pair cluster *c* that are closest to the p-arm telomere. Let *R*(*q*) denote the set of those reads in *c* that are closest to the q-arm telomere. Assume that the reads in *R*(*p*) are the mates of the reads in *R*(*q*). Thus, the set *S*={*R*(*p*)∪*R*(*q*)} is a discordant read pair cluster that supports a putative deletion, and |*R*(*p*)|=|*R*(*q*)|. Let *S*(*R*(*p*)) be a function that returns any soft-clipped reads that map to a coordinate in the range [*min*(*R*(*p*)),*min*(*R*(*p*))+*k*∗*stdev*] for *R*(*p*), where *min* returns the mapping coordinates with the lowest value among all reads in *R*(*p*). Let *S*(*R*(*q*)) be a function that returns any soft-clipped reads that map to a coordinate in the range [*max*(*R*(*q*))−*k*∗*stdev,max*(*R*(*q*))] for *R*(*q*), where *max* returns the mapping coordinates with the highest value among all reads in *R*(*q*). For all *x*∈*S*(*R*(*p*)) and *y*∈*S*(*R*(*q*)), let *mapped*(*x*) and *mapped*(*y*) denote the mapping locations of the *aligned* portion of soft-clipped reads *x* and *y*. After realigning with BLAT, let *clip*(*x*) and *clip*(*y*) denote the aligned positions of the *clipped* portion of the soft-clipped reads *x* and *y*.

#### Algorithm

The Pegasus algorithm is provided in the Algorithm 1 table.



The algorithm works by predicting the precise boundary of the SVs by observing the location of the reference where the clipped subread realigns. There may be several clipped sequences that realign to the region of a structural variant. Due to small sequence polymorphisms, it’s possible that all of the sequences may not precisely align to the same location. Thus, the predicted breakpoint within the cluster region is the one to where most of the clipped subreads align.

For all *x*∈*S*(*R*(*p*)) and *y*∈*S*(*R*(*q*)), let *mapped*(*x*) and *mapped*(*y*) denote the mapping locations of the *aligned* portion of soft-clipped reads *x* and *y*. After realigning with BLAT, let *clip*(*x*) and *clip*(*y*) denote the aligned positions of the *clipped* portion of the soft-clipped reads *x* and *y*. To predict precise deletion breakpoints, the algorithm first constructs two sets *A* and *B*, where *A*={*mapped*(*x*):∀*x*∈*S*(*R*(*p*))}∪{*clip*(*x*):∀*x*∈*S*(*R*(*p*))} and *B*={*mapped*(*y*):∀*y*∈*S*(*R*(*q*))}∪{*clip*(*y*):∀*y*∈*S*(*R*(*q*))}. To predict the deletion boundary coordinate near the reads *R*(*p*), the method returns *mode*(*A*), which gives the coordinate that occurs most often. To predict the deletion boundary near the reads *R*(*q*), the method returns *mod*(*R*(*q*)). If no breakpoint refinement can be performed (i.e. if *S*(*p*)=*∅* and *S*(*q*)=*∅*), then the algorithm will not predict the region as a deletion. Figure 1 provides an overview of the steps taken by Pegasus.

## Results

We conducted two experiments to measure the efficacy of Pegasus’ deletion prediction ability. For the first experiment, we compared our algorithm to Delly version 0.7.2 and BreakDancer version 1.1.2. Like Pegasus, Delly predicts structural variants by taking into account the mapping of paired reads and local split read alignments, while Breakdancer only considers discordant alignments of paired reads. The first experiment used simulated data created from the human reference genome to test the ability of each method to detect deletion breakpoints and to accurately predict the specific location of the breakpoints. For the second experiment, Pegasus, Delly, and Breakdancer were applied to a cancer dataset to find somatic deletions that were validated in the original study. For both experiments, the insert size cutoff was set to 4 for all three programs. For Delly, its small indel detection was turned off since all deletion variants in both datasets were greater than 1000 base pairs. Furthermore, for Delly, BreakDancer, and Pegasus, we required there to be at least 3 discordant read pairs that support a putative deletion. For Pegasus, BLAT was used for both experiments to realign the clipped portion of soft-clipped reads (the soft clipped subreads had to be at least 20 base pairs in length). For each method, the minimum read mapping quality was set to 30. For BLAT, all default parameters were used.

Regarding performance metrics, for each dataset, we compared each algorithm’s measurements on the following quantities:

**Sensitivity (SE):** the percentage of true deletion events that were correctly predicted by the algorithm.

**Average Breakpoint Error (ABE):** for each correctly predicted deletion, this is the average difference in base pairs between the true breakpoint coordinates and the predicted breakpoint coordinates. A small ABE value indicates accurate breakpoint coordinate prediction.

### Experiment 1: simulated data

For the simulated dataset, 2500 synthetic deletion variants were inserted into the human reference genome hg38 using SVSim [18]. The reads were created using Wgsim from the genome containing the synthetic deletions, and the size for the simulated events ranged from 1000 to 100,000 base pairs. BWA was used to align the reads to the reference genome hg38. The subsequent SAM/BAM file was then analyzed by both Pegasus, Delly, and BreakDancer. The results were compared by measuring the sensitivity (SE) and average breakpoint error (ABE) of each (defined above). This data had sequence read coverage of 20X and 100 base pair (bp) reads. The average insert size was 400 bp with a standard deviation of 50. The mutation rate was set to 0.001, and of those mutations, approximately 15% were indels. We created a second simulated dataset by randomly downsampling the alignments in the original BAM file, thus giving a second dataset with expected coverage of 5X. The algorithms were tested on both datasets with the same parameters.

### Experiment 2: primary prostate cancer data

The prostate cancer dataset utilized for the second experiment was from a patient (PR-0508) whose genome was analyzed in [19]. The Picard suite was used to deduplicate the alignments, after which it was aligned to the human reference genome hg18 by BWA. In order to preserve consistency hg18 was used, due to the original coordinates having been presented in this older version of the reference genome. The SAM file was analyzed by Pegasus, BreakDancer, and Delly, with comparisons being made respective to the same categories as those of the first experiment.

## Discussion

**Fig. 2:**
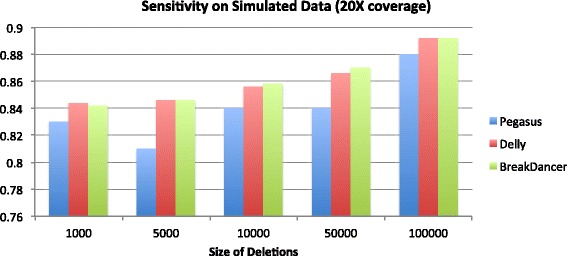
Sensitivity of predictions on 2500 simulated deletions: 20X coverage dataset. The x-axis gives the size of the deletions in base pairs (bp). There were 500 deletions per size category

**Fig. 3:**
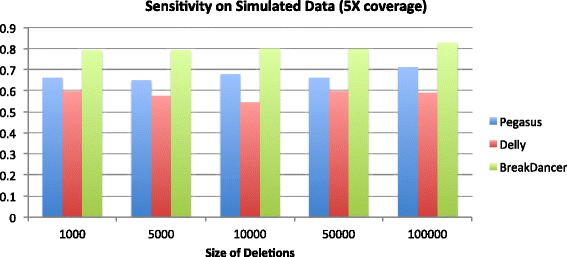
Sensitivity of predictions on 2500 simulated deletions: 5X coverage dataset

**Fig. 4:**
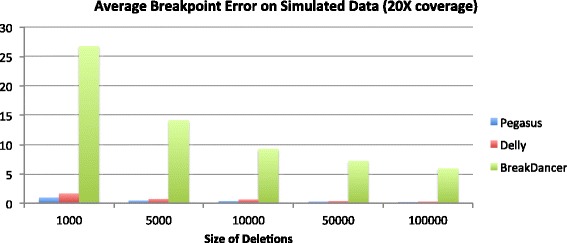
Average breakpoint error on 2500 simulated deletions: 20X coverage dataset

**Fig. 5:**
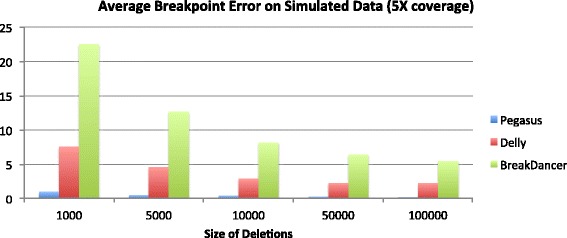
Average breakpoint error on 2500 simulated deletions: 5X coverage dataset

The results on the simulated dataset are summarized by Figs. 2, 3, 4 and 5 below. On the 20X coverage data, it can be seen in Fig. 2 that Delly and Pegasus had higher sensitivity (SE), although Pegasus also maintained a sensitivity >80% on all deletion sizes. On the lower coverage 5X dataset, however (Fig. 3), Pegasus outperformed Delly in accurately calling the deletions, though it did not outperform BreakDancer. However, in both simulated datasets, Pegasus demonstrated a lower average breakpoint error (ABE) than Delly and BreakDancer, as shown in Figs. 4 and 5.

Regarding the prostate cancer dataset, there were 22 somatic deletions reported in this sample. Delly and BreakDancer had greater sensitivity (SE=1) than Pegasus (SE=0.95), though all methods had greater overall sensitivity for the real dataset when compared to the simulated data results. Pegasus once again displayed a lower average breakpoint error (ABE=0.95) than Delly (ABE=1.02) and BreakDancer (ABE=64.6). Precise identification of breakpoints is significant in targeting specific genomic regions for therapy.

For the simiulated data, Pegasus had lower sensitivity than Delly and BreakDancer on the 20X coverage dataset, and lower sensitivity than BreakDancer on the 5X dataset. This can be partly explained by the our algorithm’s requirement for calling a deletion event. It requires 1) a cluster of discordant read pairs and 2) soft-clipped reads that map in the region of the cluster. While the second requirement ensures accurate breakpoint coordinate predictions (hence its superior performance on the ABE measurement), it also causes some events to be missed if the discordant read pairs are not proximal to soft-clipped reads, which may be due to fluctuations in sequence coverage. While relaxing the SV calling criteria would yield higher sensitivity, it would also result in higher breakpoint prediction error. Although Delly performs split-read analysis to refine its breakpoint predictions, it is not a required step of their algorithm for calling structural variants (i.e. Delly can call SVs using only paired reads). As a method based only on paired-read mapping, BreakDancer also outperformed Pegasus in terms of sensitivity. However, the consequence is that BreakDancer does not predict precise breakpoint coordinates at high accuracy, hence its very large ABE measurement across all datasets.

## Conclusions

We have presented a method to detect genomic deletions at base-pair level. Pegasus outperformed Delly and BreakDancer in predicting deletion breakpoint coordinates, while showing the ability to predict deletion events at high percentages. Regarding future work and features that will be added to Pegasus, it is not currently suited for discovering small structural variants or indel polymorphisms, which can also be important markers for cancer diagnostics and therapy. SV breakpoints have also been known to contain small microhomologies which can obfuscate SV detection and precise variant analysis [20]; Pegasus in its current state may fail to call variants near microhomology sites due to the uncertainty of breakpoint locations and clipped read mapping. Pegasus will also be extended to classify deletions as homozygous or heterozygous. Such an analysis would require examination of read depth at the location of deletions. Lastly, Pegasus will be extended to allow for filtering germline variants from SV predictions.
